# Fun at Fifty

**Published:** 1889-09-28

**Authors:** 


### The Doctor's Pulpit.

### FUN AT FIFTY.

"I am losing my sense of fun, and I am greatly concerned about it." The speaker was fifty, and was possessed of a varied experience and knowledge of the world. A man must expect to be different at fifty from what he was at thirty; but is it inevitable that he cease to enjoy fun himself and to make it for others? Is the sense of fun the same as the sense of humour? And if not, does the sense of humour develop as age advances; and is that sufficient compensation for the diminishing sense of fun? These are practical questions, at least, from the point of view of one who believes, as the writer does, that happiness is an important factor in life. Carlyle poured contempt upon those who whined for happiness, and insisted that they should be content with "blessedness" instead. "Blessedness is happiness," it might have been replied to the sage; and he who has it has happiness, and more.

The changes characteristic of the age of fifty are of great practical interest to the medical mind. For the philosophic doctor is a kind of earthly providence to his clients, and he never feels satisfied unless he keeps them, not only far from death's door, but in "good fettle," as the "horsey" world has it. Now, is it possible for the man of fifty to be in "good fettle;" and if he is in good fettle, will he not also be capable of enjoying genuine fun? Everyone of these questions must be answered in the affirmative, at any rate generally, and so far as the average man is concerned. But to this, as to other rules, there may be many exceptions.

Fifty, it must be remembered, is a temperate age. The effervescence has gone from the champagne to some extent; "the hey-day in the blood is tame, it's humble, and waits upon the judgment." But though fifty is a temperate age it is not a feeble age, neither is it an age of intellectual dullness. The man of fifty who is of a naturally robust constitution, and who has spent all his life under favourable physiological conditions, is at the very height and majesty of his intellectual strength. For that reason, if for no other, he should be capable of a great variety of fun. Some persons, however, have not in their previous lives fulfilled all the conditions of that perfect robustness which is characteristic of normal humanity at fifty. As with their wealth, so with their physiological resources, they may have spent too freely in early years. And just as such persons find that wasted wealth does not return, however wisely they think they could now use it, so physiological wealth, having once taken to itself wings, is not easily coaxed back again. Similarly, they find out that having squandered what might have increased to a considerable income, they must now accommodate themselves as best they can to straitened means. It is exactly so with the energies of health when people have been wasteful of it in youth. There are no stored resources; and life must be lived from hand to mouth. What wonder is it that under these circumstances the pinch of privation should be felt physiologically as well as financially?

What then? Must such people never expect to feel well and happy any more? Well and happy they may certainly be, but in a temperate way. Overflowing with life on all sides, and bursting with fun, they must not expect to be. Life to a cultured man or woman is a skilled art, not a mere haphazard game of shuttlecock. Goethe made it so, and with much success. We are not setting up Goethe as the brightest of all models any more than we would set up Thomas Carlyle; but Goethe had the merit of at least consuming a good deal of his own smoke, and found great advantage in so doing. It must be remembered that there are degrees of ill, and that every colic and dyspeptic depression of soul is not to be reckoned as a physiological tragedy. Besides, as age advances, at any rate up to a certain point, the nervous system becomes more sensitive and more acutely conscious of every physical ill, whilst the functional activity of the liver, and stomach, and other organs gradually becomes less energetic. So that if art does not come in as a moderator, the cultured man is likely to have a bad time of it. By art is not meant necessarily the prescriber's art, though that it is not to be despised, but the art which each man can largely acquire for himself, of understanding and managing his own constitutional and acquired peculiarities.

Now let every student of fifty bear this in mind, that the agricultural labourer and the peasant know nothing of the troubles that afflict the cultured. They have their troubles; but these are not depression of spirits, loss of the sense of fun, and inactivity of the liver. The country labourer who is prosperous, and sees his way to keeping out of the poorhouse in his old age, is decidedly a happy man, and can on adequate bucolic occasions be adequately funny. Then the question is, can anything practical be learnt from the agricultural labourer; and if so, who is to learn it? Is it enough for the doctor to study the question and to make known the results to his patient? Must not the patient himself rather take the subject in hand, and must he not resolve to apply to his own case all those well-understood principles which underlie the healthy, sleep-producing, strength-giving habits of the poor man?

The misfortune is that we all want to be well and happy in our own way, whilst Nature insists that we shall only be well and happy in hers. The mind and the circumstances are often in dire conflict; but it is only in reconciliation that quiet and happiness are to be found. There are certain departments of human activity in which every man is compelled to understand that there is such a principle as the doctrine of Necessity. "Thou shalt" and "thou shalt not" are imperatives; "thou must" and "thou must not" are inexorables. Men hate imperatives; but when they are confronted by inexorables they foam at the mouth. Now that is ridiculous in judgment and futile in act. The indifference of Nature to irrational opposition is absolute. "Whosoever shall fall on this stone shall be broken; but on whomsoever it shall fall, it shall grind him to powder." Many a Wolsey's heart, many a Henry's pride have been thus pulverised; and unto the end of time history will continue to record new examples of human obstinacy, human futility, and Nature's impassability.

Docility is the note of the highest culture; docility in action as well as in speech; docility all round. It is an "excellent oil" that lubricates all life's stiffest machinery and reduces friction to a minimum. Fun is healthy life in brisk effervescence. Can a man be really docile throughout every department of life? If so, he can always preserve the sense of fun. But many people fail to understand what docility is. They think it is mere intellectual assent. What teacher would care for such docility as that? He wants the learner to understand and approve in his heart, and to practice perfectly with his hands all that he understands and approves. How many men give full effect to all their intellectual convictions? Not one in a million. If they did, we should not have an age filled with weak complaints and childish discontents, but a world of lusty song, of proved confidence, and of high success. Men live by the old instead of living in the new. They put themselves into all sorts of conventional fetters instead of claiming the freedom they loved when children. The major part of the difficulties of life consists in this, that men insist upon fighting nineteenth century battles in ninth century armour. It cannot be done; and we constantly see all sorts of insignificant persons vanquishing all sorts of eminences and high mightinesses by the simple expedient of fighting the battles of the times with the weapons of the times. This age demands a physiological equipment of knowledge, and a physiological discipline of obedience. He who has these, other things being equal, will not be lacking in "fun at fifty."

